# Short-Term Clinical Outcomes of Transcatheter Aortic Valve Replacement in a Developing Country

**DOI:** 10.7759/cureus.58334

**Published:** 2024-04-15

**Authors:** Nadia Chamoun, Jennifer Jdaidani, Dounia Z Iskandarani, Sarah Ghalayini, Ali Zgheib, Alessandro Khoury, Samir Alam, Abdallah G Rebeiz, Ziyad Ghazzal, Fadi Sawaya

**Affiliations:** 1 Cardiology, American University of Beirut Medical Center, Beirut, LBN

**Keywords:** neurologic complications, morbidity and mortality, pacemaker insertion, third world country, developing country, clinical outcomes, aortic valve disease, tavr, transcatheter aortic valve replacement

## Abstract

Introduction: Transcatheter aortic valve replacement (TAVR) is an effective alternative to surgical aortic valve replacement (SAVR) in patients with severe aortic stenosis in all surgical risk groups. Reports of clinical outcomes post-TAVR in developing countries are scarce. We aimed to address the clinical outcomes and safety profile of TAVR in a developing country.

Methods: We conducted a single-center, retrospective study on patients undergoing TAVR at the American University of Beirut Medical Center (AUBMC) from January 2016 to April 2023. We included a total of 399 patients. Our primary endpoint was to assess the rate of TAVR in-hospital and 30-day mortality, neurologic events, and new permanent pacemaker implantation (PPI) in patients, stratified by the Society of Thoracic Surgeons (STS) risk of mortality score.

Results: Survival rates were 98.7% (394) at discharge vs. 97.5% (389) at 30 days post-procedure. The technical success rate was 95% (379) at the end of the procedure. Device success and early safety rates were 93.5% (373) and 83% (331), respectively at 30 days post-procedure. The all-cause mortality rate increased from 1.3% (5) at discharge to 2.5% (10) at 30-day intervals. The rate of ischemic stroke was 1.3% (five) at discharge and increased to 2% (eight) at 30 days post-procedure. PPI was needed in 5.8% (23) of patients at discharge with an increase to 7% (28) at one-month interval. Overall, the rates of TAVR outcomes among the three risk groups were comparable including neurologic events, valve-related complications, bleeding problems, vascular and access-related complications, and myocardial infarction.

Conclusion: This study at AUBMC highlights the successful implementation of the TAVR program in a developing country, showcasing its efficacy and safety within 30 days post-operation, despite challenges such as financial constraints and limited access to specialized training. Larger cohorts and longer follow-up periods are needed to accurately represent clinical outcomes in developing countries.

## Introduction

Transcatheter aortic valve replacement (TAVR) is considered one of the most significant breakthroughs in the field of interventional cardiology and has become the percutaneous alternative to valve surgery in patients with severe aortic stenosis (AS) over the past 20 years. In 2012, TAVR was indicated for inoperable or high-risk patients with severe AS [1]. In the following years, its use was expanded to include patients in the intermediate- and low-risk groups [1-3]. Randomized clinical trials (RCTs) [4,5] showed the clinical outcomes with TAVR to be non-inferior or superior to surgical aortic valve replacement (SAVR) across all surgical risk profiles. Moreover, valve-in-valve (ViV) TAVR for failed aortic bioprosthesis was associated with a lower risk of in-hospital mortality compared to redo-SAVR, and non-inferior when it comes to long-term outcomes [3,6,7].

While developing countries account for 70-80% of the world’s population, the leading RCTs have been centered on developed countries where the overall healthcare system differs significantly from the rest of the world. The latter’s constraints to quality healthcare are outlined by the region’s political stability, equipment availability, healthcare professionals’ skills and training, and patients’ socioeconomic status. These shortfalls render it more challenging to achieve early and adequate diagnosis and management of aortic valve disease.

Insufficient training opportunities for healthcare professionals in developing nations present a significant challenge when it comes to TAVR in these areas. Training programs specific to TAVR may be scarce or inaccessible, leading to a limited number of experts with the necessary proficiency to perform the procedure safely and effectively. The absence of comprehensive training can delay the adoption of TAVR in these countries, leaving patients with fewer treatment options and potentially compromising their health outcomes.

Additionally, financial constraints and healthcare system limitations in developing countries contribute to the challenges surrounding TAVR. The cost of the procedure, including the equipment, prosthetic valves, and follow-up care, can be prohibitively high for many patients and healthcare systems with limited resources. The lack of adequate reimbursement mechanisms and health insurance coverage further exacerbates the financial barriers to accessing TAVR in these settings.

In the absence of sufficient data in developing countries, including Lebanon, we sought to report the in-hospital and 30-day clinical outcomes of TAVR at the American University of Beirut Medical Center (AUBMC), the largest referral center in Lebanon. It is important to note that the true safety and efficacy of such a complex procedure can only be assessed by real-world data, particularly in developing countries where healthcare resources and patient profiles may differ significantly. Moreover, while conducting our study, we encountered limited available data on TAVR outcomes within Lebanon, with only one study of 141 patients conducted at Hotel Dieu de France Hospital [8]. This highlights the need for additional research and data collection in Lebanon to provide a comprehensive understanding of TAVR outcomes and enable evidence-based decision-making in this context.

## Materials and methods

Objectives

Primary

Our primary objective was to assess TAVR in-hospital and 30-day mortality, neurologic events, and new permanent pacemaker implantation (PPI) in patients, stratified by the Society of Thoracic Surgeons (STS) risk of mortality score, from January 2016 to April 2023.

Secondary

Our secondary objective was to assess TAVR in-hospital and 30-day bleeding, vascular complications, cardiac and valve-related complications, new-onset conduction disturbances and arrhythmias, acute kidney injury (AKI), myocardial infarction (MI), and procedure-related re-hospitalization in patients, stratified by STS risk of mortality score.

Composite Endpoints

These include technical success at procedure exit as well as 30-day device success and early safety.

Material and methods

Study Design and Setting

We conducted a single-center, retrospective study on patients undergoing TAVR at AUBMC. We retrieved data from the TAVR registry of the structural heart program at AUBMC, cardiology division, from January 2016 to April 2023. We followed up with patients for a duration of 30 days post-TAVR.

Participants

We included all patients undergoing TAVR at AUBMC in the study, irrespective of their risk score. We evaluated the patients’ surgical risk based on the STS risk of mortality score. We stratified patients into three groups: those with an STS score ≥8% were classified as high risk, a score of 4-8% as intermediate risk, and a score <4% as low risk.

Patients eligible for TAVR underwent the procedure following approval from the structural heart program comprising an interventional cardiologist and a cardiothoracic surgeon. We included in this study patients with symptomatic severe AS, aortic regurgitation (AR), or bio-prosthetic structural valve deterioration (SVD). We defined severe AS as an aortic valve area (AVA) of ≤1 cm^2^ or ≤0.6 cm^2^/m^2^, a mean aortic-valve pressure gradient (MG) of ≥40 mmHg, or a peak aortic-jet velocity (Vmax) ≥4.0 m/s, as measured by transthoracic echocardiography (TTE), or patients with low-flow, low-gradient symptomatic AS with demonstrated high aortic valve leaflet calcification scores. We defined severe AR as regurgitation volume ≥60 mL/beat and/or regurgitation fraction ≥50% and/or effective regurgitant orifice area (EROA) ≥0.3 cm^2^. We defined severe SVD as valve-related dysfunction (MG) ≥20 mmHg, effective orifice area (EOA) ≤0.9-1.1 cm^2^, and/or dimensionless valve index (DVI) <0.35, and/or moderate or severe prosthetic valve regurgitation requiring the need for a repeat procedure (TAVR or SAVR) [9].

Variables

We collected demographics, past medical histories, pre- and post-TAVR workups, procedural characteristics, and clinical outcomes of patients from the TAVR registry. Pre-TAVR workup included routine blood tests, electrocardiogram (ECG), TTE, transesophageal echocardiography (TEE) if needed, selective coronary angiography, and computed tomography (CT) TAVR protocol. We used CT TAVR as the standard method to evaluate the aortic root anatomy and confirm suitability for a femoral approach. Valve choice was based on physician preference tailored to the CT analysis and patient characteristics; valves used were either balloon-expandable valves (Sapien XT/S3 (Irvine, California, United States), Myval (Meril, India)) or self-expandable valves (Medtronic Evolut R/PRO (Minneapolis, Minnesota, United States), Abbott Vascular Portico/Navitor (Abbott Park, Illinois, United States), Boston Scientific Lotus Edge, Acurate Neo/Acurate Neo 2 (Marlborough, Massachusetts, United States)). With five types of valves available at our center, we customized our approach based on the patient's age and the specific anatomical details revealed by CT scans. In younger patients, we often opted for a balloon-expandable or intra-annular valve. For patients with small annuli, we typically chose a self-expandable supra-annular platform, primarily for its superior hemodynamics. In cases of highly calcified valves or severe left ventricular outflow tract (LVOT) calcification, we preferred the Medtronic platform due to its good radial force properties and lower risk of rupture. When treating bicuspid valves, we selected either the Medtronic or balloon-expandable platform, based on the length of the raphe and the extent of calcification. Thus, the selection of the valve platform was carefully customized to meet the specific needs of each patient and their anatomical characteristics.

Post-TAVR workup included routine blood tests, ECG, and TTE. Technical success was characterized as the immediate outcome of the procedure, assessed upon exiting the procedure room. This metric primarily focused on the actual technical safety of the device and its deployment. The criterion for device success considered issues related to the procedure or valve that emerged post-technical success, while also taking into account the initial performance of the valve. We collected the TAVR clinical outcomes and composite endpoints according to the latest VARC-3 criteria for aortic valve clinical research published in April 2021 [10].

Statistical Methods

We managed data and analyzed statistics using the Statistical Package for the Social Sciences (IBM SPSS Statistics for Windows, IBM Corp., Version 27.0, Armonk, NY). All tests were two-tailed and a p-value <0.05 was considered significant. We performed a descriptive analysis, stratified per STS, for the variables mentioned above. We reported categorical variables as percentages (%) and frequencies (n) and continuous variables as mean (standard deviation) or median (IQR). We conducted a bivariate analysis to compare individual variables between the different surgical risk groups. We used Chi-square or Fisher’s test to compare categorical variables. As for continuous variables, we established normality using a histogram and applied a one-way analysis of variance (ANOVA) or Kruskal-Wallis test accordingly.

Ethics Committee Information

The institutional review board (IRB) at AUBMC reviewed and approved this study. We obtained informed consent from the enrolled patients as part of the TAVR registry inclusion protocol. The study protocol conformed to the ethical guidelines of the 1975 Declaration of Helsinki.

## Results

**Figure 1:**
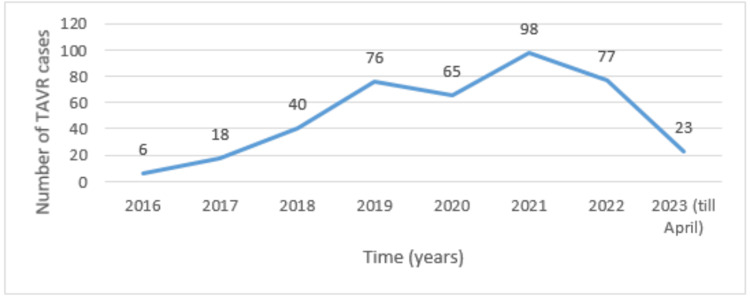
Number of TAVR cases per year TAVR: transcatheter aortic valve replacement

A total of 399 patients who underwent TAVR at our institution from January 2016 to April 2023 were included in this study. The mean age of the population was 81 (75-85) with 52.1% (208) being male. Most of the population belonged to the low-risk group (50.1% (200)), followed by the intermediate-risk group (34% (136)), and high-risk group (15.8% (63)). Refer to Figure 1 for the number of TAVR cases per year.

Patients baseline characteristics and pre-procedural laboratory and echocardiography results

Tables 1-2 summarize the baseline characteristics, pre-procedural laboratory, and echocardiogram results of TAVR patients stratified by their STS risk score.

**Table 1:** Baseline characteristics of TAVR patients stratified according to patient STS risk score (low, intermediate, and high-risk) Continuous variables are presented by median (IQR); categorical variables are presented by count (%). TAVR: transcatheter aortic valve replacement; STS: Society of Thoracic Surgeons; TIA: transient ischemic attack; RBBB: right bundle branch block; LBBB: left bundle branch block; NYHA: New York Heart Association; CAD: coronary artery disease; PCI: percutaneous coronary intervention; CABG: coronary artery bypass graft; PM: pacemaker; AV: aortic valve; SMVR: surgical mitral valve replacement; NSTEMI: non-ST elevation myocardial infarction; STEMI: ST elevation myocardial infarction; CHF: congestive heart failure

Variables	Patients’ STS score				
	All (n=399)	Low risk (n=200)	Intermediate risk (n=136)	High risk (n=63)	P-value
Demographics					
Age (Years)	81 (75-85)	79 (74-84)	81 (76-85)	83 (79-89)	<0.001
Gender (Male)	208 (52.1)	119 (59.5)	63 (46.3)	26 (41.3)	0.01
Body mass index (kg/m^2^)	28.36 (24.64-31.5)	29 (26-31)	27.9 (23.8-31.25)	26.9 (23-32)	0.033
Smoking status (Ever smoker)	145 (36.3)	76 (38)	55 (40.4)	14 (22.2)	0.036
Past Medical History					
Hypertension	345 (86.5)	169 (84.5)	117 (86)	59 (93.7)	0.17
Diabetes mellitus	156 (39.1)	89 (44.5)	45 (33.1)	22 (34.9)	0.083
Hyperlipidemia	261 (65.4)	137 (68.5)	86 (63.2)	38 (60.3)	0.4
Chronic lung disease	113 (28.3)	46 (23)	44 (32.4)	23 (36.5)	0.051
Chronic kidney disease	124 (31.1)	44 (22)	52 (38.2)	28 (44.4)	<0.001
On dialysis	9 (2.3)	0	5 (3.7)	4 (6.3)	0.001
Cerebrovascular disease (Stroke/TIA)	27 (6.8)	12 (6)	8 (5.9)	7 (11.1)	0.33
Peripheral artery disease	53 (13.3)	22 (11)	19 (14)	12 (19)	0.3
Carotid artery stenosis	17 (4.3)	6 (3)	7 (5.1)	4 (6.3)	0.36
Past Cardiac History					
RBBB	33 (8.3)	18 (9)	11 (8.1)	4 (6.3)	0.8
LBBB	55 (13.9)	25 (12.6)	20 (14.8)	10 (15.9)	0.75
Atrial fibrillation	127 (31.8)	55 (27.5)	47 (34.6)	25 (39.7)	0.13
Porcelain aorta	21 (5.3)	13 (6.5)	5 (3.7)	3 (4.8)	0.56
NYHA III	282 (70.9)	152 (76.4)	99 (72.8)	31 (49.2)	<0.001
NYHA IV	104 (26.1)	36 (18.1)	36 (26.5)	32 (50.8)	<0.001
CAD	119 (29.9)	62 (31.2)	40 (29.4)	17 (27)	0.8
PCI	114 (28.6)	53 (26.5)	45 (33.1)	16 (25.4)	0.35
CABG	84 (21)	34 (17)	32 (23.5)	18 (28.6)	0.1
PM	28 (7)	16 (8)	2 (1.5)	10 (15.9)	<0.001
AV valvuloplasty	5 (1.3)	4 (2)	0	1 (1.6)	0.3
SMVR	12 (3)	4 (2)	5 (3.7)	3 (4.8)	0.4
Pre-procedural Clinical Status					
NSTEMI (<7 days)	3 (0.8)	0	2 (1.5)	1 (1.6)	0.16
STEMI (<7 days)	1 (0.3)	0	1 (0.7)	0	0.5
CHF exacerbation (<14 days)	94 (23.6)	28 (14)	36 (26.5)	30 (47.6)	<0.001

**Table 2:** Pre-TAVR laboratories and echocardiography results stratified by STS risk score Continuous variables are presented by median (IQR); categorical variables are presented by count (%). TAVR: transcatheter aortic valve replacement; STS: Society of Thoracic Surgeons; LVEF: left ventricular ejection fraction; HFrEF: heart failure with reduced ejection fraction; AV: aortic valve; PPG: peak pressure gradient; MPG: mean pressure gradient, SPAP: systolic pulmonary artery pressure *Mixed aortic valve disorder is defined as severe aortic stenosis with an aortic valve regurgitation grade of ≥3.

Variables	Patients’ STS score				
	All (n=399)	Low risk (n=200)	Intermediate risk (n=136)	High risk (n=63)	P-value
Pre-procedural Laboratories					
Hemoglobin (g/dL)	11.7 (10.4-12.9)	12.2 (11-13.3)	11.3 (10-12.5)	11 (9.7-11.8)	<0.01
Creatinine (mg/dL)	1 (0.8-1.3)	1 (0.8-1.1)	1.1 (0.8-1.4)	1.2 (1-1.9)	<0.01
Pre-procedural Echocardiography					
LVEF %	60 (50-65)	60 (55-65)	60 (50-60)	60 (40-65)	0.377
HFrEF	84 (22)	36 (19.7)	27 (20)	21 (33.9)	0.051
Area (cm^2^)	0.75 (0.6-0.87)	0.75 (0.62-0.85)	0.78 (0.6-0.87)	0.75 (0.6-0.88)	0.96
AV PPG (mmHg)	70.5 (57-81.2)	71 (59-82.5)	71 (56-82)	64 (56-80)	0.348
AV MPG (mmHg)	44 (37-53)	45 (40-54)	44 (36-51)	40 (34.5-50)	0.08
SPAP (mmHg)	42 (37-53)	37 (30-50)	46 (36-58)	54 (42-68)	<0.001
Mitral regurgitation ≥3	35 (9.6)	10 (5.6)	15 (11.5)	10 (16.9)	0.024
Tricuspid regurgitation ≥3	49 (14.2)	9 (5.6)	19 (15.1)	21 (36.8)	<0.001
Aortic Valve Disease					
Isolated AV stenosis	344 (87.3)	180 (91.8)	116 (85.9)	48 (76.2)	0.02
Isolated AV regurgitation	21 (5.3)	9 (4.6)	6 (4.4)	6 (9.5)	0.29
Mixed AV disease*	29 (7.4)	7 (3.6)	13 (9.6)	9 (14.3)	0.005
Aortic Valve Morphology					
Tricuspid AV	355 (89)	181 (90.5)	120 (88.2)	54 (85.7)	0.54
Bicuspid AV	7 (1.8)	5 (2.5)	2 (1.5)	0	0.6
Bioprosthetic valve	32 (8)	11 (5.5)	13 (9.6)	8 (12.7)	0.13

The high-risk group participants, compared to those in the intermediate- and low-risk groups, were older (83 (79-89); p <0.001), had a higher prevalence of chronic kidney disease (CKD) (44.4% (28); p<0.001), New York Heart Association (NYHA) Class IV (50.8% (32); p <0.001), previously inserted permanent pacemaker (15.9% (10); p<0.001), congestive heart failure (CHF) exacerbation in the last 14 days pre-procedure (47.6% (30); p <0.001), mitral regurgitation (MR) grade ≥3 (16.9% (10); p=0.024), tricuspid regurgitation (TR) grade ≥3 (36.8% (21); p <0.001), and mixed aortic valve disease defined as severe AS with an aortic valve regurgitation grade of ≥3 (14.3% (9); p=0.005). Moreover, high-risk group patients had a lower pre-operative hemoglobin (Hb) level (11 g/dL (9.7-11.8); p <0.01), a higher pre-operative creatinine (Cr) level (1.2 mg/dL (1-1.9); p <0.001) and systolic pulmonary artery pressure (SPAP) (54 mmHg (42-68); p <0.001).

Most patients who underwent TAVR presented with isolated AS (87.3% (344)), while 5.3% (21) presented with isolated AR, and 7.4% (29) had mixed AV disease. Among the 399 patients who presented for TAVR, 1.8% (7) had a bicuspid valve on echocardiography.

Procedural characteristics and post-TAVR echocardiography results

Tables 3-4 describe procedural characteristics and post-TAVR laboratory and echocardiography results stratified by STS risk score.

**Table 3:** TAVR procedural characteristics stratified by STS risk score (low, intermediate, and high-risk) Categorical variables are presented by count (%); continuous variables are presented by median (IQR). TAVR: transcatheter aortic valve replacement; SAVR: surgical aortic valve replacement; STS: Society of Thoracic Surgeons

Variables	Patients’ STS score				P value
	All (n=399)	Low risk (n=200)	Intermediate risk (n=136)	High risk (n=63)	
Approach					
Trans-femoral	388 (97.2)	196 (98)	131 (96.3)	61 (96.8)	0.5
Trans-axillary	5 (1.3)	1 (0.5)	3 (2.2)	1 (1.6)	0.3
Trans-carotid	1 (0.3)	0	0	1 (1.6)	0.42
Left subclavian artery	1 (0.3)	0	1 (0.7)	0	0.56
Trans-aortic	2 (0.5)	2 (1)	0	0	0.3
Trans-caval	2 (0.5)	1 (0.5)	1 (0.7)	0	0.326
Anesthesia					
General	10 (2.5)	4 (2)	4 (2.9)	2 (3.2)	0.748
Conscious sedation	389 (97.5)	196 (98)	132 (97.1)	61 (96.8)	
Valve-in-Valve Procedure					
TAVR-in-SAVR	30 (7.5)	11 (5.5)	11 (8.1)	8 (12.7)	0.15
TAVR-in-TAVR	2 (0.5)	0	2 (1.5)	0	0.3
Dilation					
Pre-dilation	191 (48.7)	93 (47)	65 (48.5)	33 (55)	0.56
Post-dilation	109 (27.8)	58 (29.4)	31 (23.1)	20 (32.8)	0.2
Total Procedural Time (min)	89 (60-120)	89 (61-118)	82 (60-120)	90 (60-125)	1

**Table 4:** Post-TAVR echocardiography results stratified by STS risk score (low, intermediate, and high-risk) Continuous variables are presented by median (IQR); categorical variables are presented by count (%). TAVR: transcatheter aortic valve replacement; STS: Society of Thoracic Surgeons; LVEF: left ventricular ejection fraction; AV: aortic valve; PPG: peak pressure gradient; MPG: mean pressure gradient; SPAP: systolic pulmonary artery pressure

Variables	Patients’ STS score				
	All (n=399)	Low risk (n=200)	Intermediate risk (n=136)	High risk (n=63)	P-value
Post-procedural Echocardiography					
LVEF (%)	60 (55-65)	60 (55-65)	44 (36-51)	40 (34.5-50)	0.2
AV PPG (mmHg)	15 (11-20)	16 (12-20)	14 (10-19)	15 (10-23)	0.26
AV MPG (mmHg)	8 (5-11)	9 (6-11)	7 (5-11)	7.5 (5-12)	0.24
SPAP (mmHg)	39 (32-51)	37 (30-44)	42 (33-54)	42 (35-60)	<0.001
Mitral Regurgitation ≥ 3	29 (7.5)	10 (5.1)	11 (8.2)	8 (13.6)	0.08

**Figure 2:**
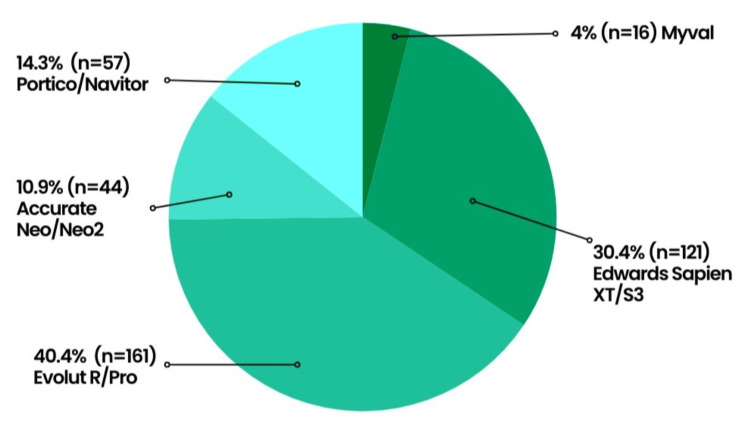
Bioprosthetic valves deployed during TAVR TAVR: transcatheter aortic valve replacement

The procedure was predominantly done under local anesthesia (95.7% (389)) and through femoral access (97.2% (388)). The ViV procedure was performed in 32 (8%) patients who previously underwent SAVR (7.5% (30)) or TAVR (0.5% (2)). The choice of prosthetic valves was not influenced by the patient’s STS risk score, with Evolut R/Pro being the most frequently used valve (40.4% (161)) (Figure 2).

Pre-dilation and post-dilation were performed in 48.7% (191) and 27.8% (109) of patients, respectively. On average, the TAVR procedure needed 89 minutes (60-120). Patients immediately post-TAVR had an average LVEF of 60% (55-65), a mean pressure gradient of 8 mmHg (5-11), and an SPAP of 39 mmHg (32-51).

Procedural and in-hospital TAVR outcomes

The procedural and in-hospital outcomes of TAVR are presented in Table 5.

**Table 5:** Procedural and in-hospital TAVR outcomes stratified by STS risk score (low, intermediate, and high-risk) Continuous variables are presented by median (IQR); categorical variables are presented by count (%). TAVR: transcatheter aortic valve replacement; STS: Society of Thoracic Surgeons; PVL: paravalvular leak; AKI: acute kidney injury; MI: myocardial infarction; LBBB: left bundle branch block; RBBB: right bundle branch block; Afib: atrial fibrillation; PPI: permanent pacemaker insertion

Variables	Patients’ STS score			
	All (n=399)	Low risk (n=200)	Intermediate risk (n=136)	High risk (n=63)
Mortality				
All-cause mortality	5 (1.3)	0	1 (0.7)	4 (6.3)
Cardiovascular	4 (1)	0	1 (0.7)	3 (4.8)
Non-cardiovascular	1 (0.3)	0	0	1 (1.6)
Neurologic Events				
Ischemic stroke	5 (1.3)	2 (1)	2 (1.5)	1 (1.6)
Periprocedural Acute	4 (1)	2 (1)	2 (1.5)	0
Periprocedural Sub-acute	1 (0.2)	0	0	1 (1.6)
Acute Procedural and Technical Valve-Related Complications				
Conversion to open surgery	1 (0.3)	1 (0.5)	0	0
Implantation of multiple (>1) transcatheter valves	4 (1)	1 (0.5)	1(0.7)	2 (3.2)
PVL Grade 1	120 (30.3)	60 (30)	41 (30.1)	19 (31.7)
PVL Grade 2	37 (9.3)	16 (8)	16 (11.8)	5 (8.3)
PVL Grade 3	7 (1.8)	2 (1)	4 (2.9)	1 (1.7)
PVL Grade 5	1 (0.3)	1 (0.5)	0	0
PVL plugs	1 (0.3)	1 (0.5)	0	0
Bleeding Complications				
Type I	5 (1.3)	3 (1.5)	2 (1.5)	0
Type II	8 (2)	2 (1)	5 (3.7)	1 (1.6)
Type III	4 (1)	4 (2)	0	0
Type IV	1 (0.3)	0	0	1 (1.6)
Cardiac Structural Complications				
Major	10 (2.5)	6 (3)	1 (0.7)	3 (4.8)
Vascular and Access-Related Complications				
Major	3 (0.8)	3 (1.5)	0	0
Minor	23 (5.8)	12 (6)	10 (7.4)	1 (1.6)
New Conduction Disturbances and Permanent Pacemaker Insertion				
LBBB	50 (12.5)	16 (8)	26 (19)	8 (12.7)
RBBB	5 (1.3)	2 (1)	2 (1.5)	1 (1.6)
Third-degree atrioventricular block	22 (5.5)	8 (4)	6 (4.4)	8 (12.7)
Afib	2 (0.5)	1 (0.5)	1 (0.7)	0
PPI	23 (5.8)	8 (4)	8 (5.9)	7 (11)
Other Outcomes				
Any AKI	45 (11.3)	12 (6)	16 (11.8)	17 (27)
AKI Stage ≥3	11 (2.8)	2 (1)	4 (2.9)	5 (7.9)
MI (<48 hours post-procedure)	4 (1)	3 (1.5)	0	1 (1.6)
Hospital Length of Stay (days)	2 (1-2)	2 (1-2)	2 (1-2)	2 (1-3)

In-hospital all-cause mortality was reported in five (1.3%) TAVR patients, two of whom were intra-procedural and three were in-hospital post-procedural deaths. Four out of the five reported deaths occurred in the high-risk group and no deaths were encountered in the low-risk group. Cardiovascular complications were responsible for four out of the five in-hospital deaths: one case of intra-procedural MI due to left main artery occlusion, one case of intra-procedural left ventricle perforation leading to cardiac tamponade, one case of post-procedural ischemic stroke, and one case of post-procedural pericardial tamponade and pulmonary embolism. The fifth in-hospital mortality was non-cardiovascular attributed to a post-procedural hospital-acquired pneumonia leading to septic shock.

In-hospital ischemic strokes were reported in five (1.3%) TAVR patients: three occurred intra-procedurally, one within the first 24 hours post-procedure, and one >24 hours post-procedure. One case of ischemic stroke led to a subarachnoid hemorrhage and death three days post-TAVR. Two of the intra-procedural strokes were treated successfully with aspiration thrombectomy.

A second valve was needed in four (1%) TAVR patients due to valve embolization, one of which required conversion to open surgery due to the risk of aortic root injury. Greater or equal to moderate paravalvular leak (PVL) (grade ≥3) was detected in eight (2.1%) patients, one of which was severe (grade 5) necessitating a vascular plug. One case of SVD was identified in a high-risk patient due to perforation of the valve leaflet post-valve dilation leading to a moderate to severe eccentric central AR. The SVD was subclinical, morphologic (stage 1) in nature necessitating no intervention.

More than minor bleeding complications (grade ≥2) occurred in 13 (3.3%) TAVR patients including eight (2%) cases of major (type II) bleeding, four (1%) cases of life-threatening (type III) bleeding, and one (0.3%) case of bleeding leading to death (type IV). The latter was a consequence of intra-procedural left ventricular (LV) perforation and tamponade.

New left bundle branch block (LBBB) and third-degree atrioventricular (AV) block were described in 12.5% (50) and 5.5% (22) of TAVR patients, respectively. After excluding those with a previous cardiac device, 5.8% (23) of TAVR patients required a PPI post-procedure.

As for vascular and access-related complications, a total of 26 (6.6%) cases were recorded, three (0.8%) of which were major cases. All three cases of major vascular events occurred in low-risk patients and required surgical intervention: one necessitating repair of a dissected external iliac artery, one requiring endarterectomy and repair of a dissected common femoral artery using a bovine pericardial patch, and one requiring surgical closure of right femoral access site due to failure of ProGlide closure.

Cardiac structural complications were reported in 2.5% (10) of TAVR patients: Three cases of post-operative left main coronary artery occlusion, two of which required stenting and one leading to death, one case of intra-operative LV perforation and tamponade leading to death, one case of LV outflow tract obstruction, one case of post-procedural pericardial tamponade and pulmonary embolism leading to death, one case of post-procedural cardiac arrest with ventricular fibrillation that required shock with the restoration of normal sinus rhythm, one case of post-operative pericardial tamponade due to perforation by RV temporal pacemaker wire with hemodynamic instability requiring pericardiocentesis, one case of intra-operative complete occlusion of a right coronary artery by calcified embolus leading to ventricular fibrillation successfully treated with defibrillation, and one case of cardiogenic shock leading to stage IV AKI.

AKI occurred in 45 (11.3%) patients post-TAVR, 11 (24.4%) of which were grade ≥ 3, and six (13.3%) patients required temporary or permanent renal replacement therapy. On average, patients were hospitalized for a duration of two days (one to two) post-procedure.

One-month TAVR outcomes

Table 6 represents the 30-day TAVR outcomes stratified by STS risk score.

**Table 6:** Thirty-day TAVR outcomes stratified by STS risk score (low, intermediate, and high-risk) Continuous variables are presented by median (IQR); categorical variables are presented by count (%). TAVR: transcatheter aortic valve replacement; STS: Society of Thoracic Surgeons; PVL: paravalvular leak; TIA: transient ischemic attack; AKI: acute kidney injury; MI: myocardial infarction; LBBB: left bundle branch block; Afib: atrial fibrillation; PPI: permanent pacemaker insertion

Variables	Patients’ STS score			
	All (n=399)	Low risk (n=200)	Intermediate risk (n=136)	High risk (n=63)
Mortality				
All-cause mortality	10 (2.5)	2 (1)	1 (0.7)	7 (11.1)
Cardiovascular	6 (1.5)	1 (0.5)	1 (0.7)	4 (6.3)
Non-cardiovascular	3 (0.8)	0	0	3 (4.8)
Valvular cause of death	1 (0.3)	1 (0.5)	0	0
Neurologic Events				
Ischemic stroke	8 (2)	4 (2)	2 (1.5)	2 (3.2)
Hemorrhagic stroke	2 (0.5)	1 (0.5)	1 (0.7)	0
TIA	2 (0.5)	1 (0.5)	0	1 (1.6)
Cardiac Structural Complications				
Major	11 (2.75)	7 (3.5)	1 (0.7)	3 (4.8)
Re-hospitalization				
Procedure or valve-related	13 (3.3)	8 (4)	3 (2.2)	2 (3.2)
Other cardiovascular	7 (1.8)	2 (1)	2 (1.5)	3 (4.8)
Non-cardiovascular	18 (4.50)	5 (2.5)	6 (4.4)	7 (11.1)
New-Onset Conduction Disturbances and Arrhythmias				
LBBB	51 (12.8)	16 (8)	27 (19.9)	8 (12.7)
Afib	4 (1)	2 (1)	1 (0.7)	1 (1.6)
Third-degree atrioventricular block	26 (6.5)	10 (5)	8 (5.9)	8 (12.7)
PPI	28 (7)	10 (5)	11 (8.1)	7 (11.1)
Other Outcomes				
AKI Stage ≥ 3	13 (3.3)	2 (1)	5 (3.7)	6 (9.5)
MI	5 (1.3)	4 (2)	0	1 (1.6)

The rate of mortality increased from 1.3% (5) in-hospital to 2.5% (10) within 30 days following the procedure due to two cardiovascular deaths, one valve-related and two non-cardiovascular causes of death: one case of ischemic stroke, one case of complete heart block with junctional escape rhythm and failure of pacemaker to capture, one case of acute valve thrombosis leading to bioprosthetic valve failure and cardiogenic shock, one case of septic shock, and one case of aspiration pneumonia, respectively.

Three additional cases of ischemic stroke were recorded post-discharge, increasing the overall rate to 2% (eight). Moreover, two cases of hemorrhagic stroke and two transient ischemic attacks (TIA) occurred during the 30-day interval after discharge. Thirty-eight (9.6%) TAVR patients required re-hospitalization within 30 days post-procedure because of 13 (3.3%) cases of a procedure or valve-related complications, seven (1.8%) cardiovascular problems, and 18 (4.5%) non-cardiovascular events. Table 7 summarizes the procedural and cardiovascular causes of re-hospitalization post-TAVR.

**Table 7:** Causes of re-hospitalization post-TAVR TAVR: transcatheter aortic valve replacement; AV: atrioventricular; Afib: atrial fibrillation; CAD: coronary artery disease

Causes of re-hospitalization	Description
Procedure or valve-related re-hospitalization (n=13)	Third-degree AV block (n=6)
	Sick sinus syndrome requiring PPI (n=1)
	Ischemic stroke (n=3)
	Loss of capture of right ventricular lead (n=1)
	Acute bioprosthetic valve thrombosis (n=1)
	Myocardial infarction (n=1)
Other cardiovascular hospitalization (n=7)	Chest pain due to non-obstructive CAD (n=2)
	Fatigue and dyspnea due to previous endocarditis (n=1)
	Congestive heart failure exacerbation (n=2)
	Supraventricular tachycardia (n=1)
	Chest pain due to paroxysmal Afib (n=1)

Additionally, one additional case of LBBB and four new cases of third-degree AV block were recorded post-discharge. One case of MI occurred in a low-risk patient raising the rate of major cardiac complications to 2.75% (11), and two cases of AKI grade ≥3 were reported post-discharge.

Permanent pacemaker (PPM) outcome

Excluding the patients who had a cardiac device (PM/ICD) inserted pre-TAVR, new PPI was required for 23 (5.8%) TAVR patients at discharge. PPMs were inserted because of new conduction disturbances including third-degree AV block (18 cases), wide LBBB > 160 mm (three cases), sick sinus syndrome (SSS) (one case), and trifascicular block (one case). Post-hospital discharge and within 30 days post-procedure, five additional PPM (total of 7% (28)) were inserted due to four cases of third-degree AV block and one case of SSS.

**Figure 3:**
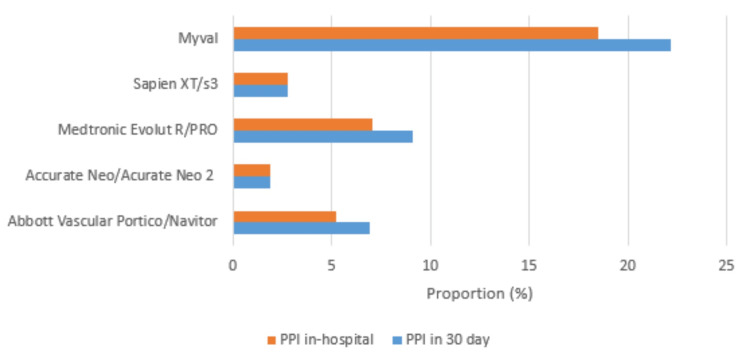
Proportion of new permanent pacemaker insertion stratified by the type of bioprosthetic valve used PPI: permanent pacemaker insertion

The proportion of TAVR patients requiring new PPI at discharge and at one-month intervals was not influenced by the choice of bioprosthetic valve. However, this rate was numerically lowest in the Acurate Neo/Acurate Neo 2 group compared to the other valve types, at both intervals (Figure 3).

Composite endpoints

Composite endpoints including intra-procedural technical success and 30-day device success and early safety, stratified by STS score, were documented in Table 8.

**Table 8:** Composite endpoints including technical success (at exit from procedure room), device success at 30-day procedure and 30-day early safety, stratified by STS risk score (low, intermediate, and high-risk) Categorical variables are presented by count (%). STS: Society of Thoracic Surgeons

Variables	Patients’ STS score				
	All (n=399)	Low risk (n=200)	Intermediate risk (n=136)	High risk (n=63)	P-value
Composite endpoints					
Technical success (at exit from procedure room)	379 (95)	188 (94)	134 (98.5)	57 (90.5)	0.023
30-day device success	373 (93.5)	185 (92.5)	133 (97.8)	55 (87.3)	0.011
30-day early safety	331 (83)	171 (85.5)	117 (86)	43 (68.3)	0.003

Technical success at the conclusion of the procedure and device success at 30 days were 95% (379) and 93.5% (373) respectively, with higher rates in the low- and intermediate-risk groups compared to the high-risk group. Moreover, 30-day early safety was achieved in 83% (331) of TAVR patients with comparable rates in the low- and intermediate-risk groups (85.5% (171) and 86% (117), respectively), and higher than that reported in the high-risk group (68.3% (43)).

## Discussion

To the best of our knowledge, our study represents one of the largest single-center datasets on TAVR in developing countries. It demonstrated high success rates with low early complication rates, comparable to those reported in developed countries [1,2,11,12]. Notably, the survival rates were 98.7% (394 patients) at discharge and 97.5% (389 patients) at 30 days post-procedure. Technical success at the end of the procedure was achieved in 95% (379 patients) of our cases. Additionally, device success and early safety at 30 days were recorded for 93.5% (373 patients) and 83% (331 patients) of TAVR patients, respectively. The 30-day mortality rate in our study was found to be lower than that reported in the literature from developed countries [13]. In comparison, Bahaa et al. reported an in-hospital and 30-day mortality rate of 4.16% in their cohort in Egypt [14]. While our rates were lower, the findings from Bahaa et al. still demonstrate a high rate of survival, underscoring the effectiveness of TAVR in developing countries. Both studies illustrate that despite the financial and infrastructural challenges prevalent in these regions, TAVR can be successfully implemented, providing substantial benefits with mortality rates and technical successes that closely align with global standards.

Most TAVR procedures were performed in patients belonging to the low-risk group (50.1% or 200 patients), followed by intermediate-risk (34% or 136 patients) and high-risk groups (15.8% or 63 patients). Being in the low-risk category does not necessarily mean being young. Our low-risk group had a median age of 79 years (range 74-84) and a high prevalence of several comorbidities such as hypertension (HTN), diabetes mellitus (DM), hyperlipidemia, and CKD. Additionally, the STS score does not account for other indices like frailty and porcelain aorta, which are critical. The high-risk group exhibited the highest prevalence of several comorbidities such as CKD, hemodialysis, CHF, and previous PPM, comparable to other studies [15]. In line with another study [15], an intriguing finding in our paper is the high procedural success rate observed irrespective of the risk group, with most procedural outcomes being similar among the three risk groups.

Neurologic events, including ischemic strokes, hemorrhagic strokes, symptomatic hypoxic-ischemic injury, and TIA, are considered one of the most critical adverse events post-TAVR. Although the rate of stroke post-TAVR has decreased with the evolution of this procedure, cerebrovascular accidents remain a significant clinical outcome with increased risk of morbidity and mortality in TAVR patients [10]. Studies conducted in developed countries have reported a rate of ischemic stroke ranging from 3% to 6% [16]. In our analysis, we recorded an overall ischemic stroke rate of 2% (eight patients). Despite the minimal use of cerebral protection devices, this relatively low rate can be attributed to the extensive workup performed prior to TAVR and the meticulous care provided during and after the procedure, including adequate antithrombotic therapy. Post-TAVR patients were prescribed a single antithrombotic medication, including antiplatelets (such as aspirin or P2Y12 inhibitors) and/or anticoagulants in patients with atrial fibrillation (such as novel oral anticoagulants like factor X inhibitors), which is reported to be crucial for the prevention of ischemic strokes post-TAVR [16].

Several studies have investigated the association between TAVR and new conduction abnormalities and arrhythmias, including LBBB, AV block, and atrial fibrillation [17]. This association is primarily related to the close anatomical relationship between the aortic valve and the heart conduction system [18]. New-onset LBBB is reported to be the most common conduction disturbance post-TAVR, with an incidence ranging between 7% and 83% [19]. In our study, the development of new-onset LBBB was relatively low among TAVR patients, with a rate of 12.8% (51 patients). Patients with new-onset LBBB post-TAVR were elderly, with a mean age of 81 years, and had a high burden of comorbidities. The incidence of LBBB post-TAVR also depends on the type of device used [17]. For instance, the incidence of LBBB ranges from 35% to 65% with the self-expanding Medtronic CoreValve system, and from 3% to 10% with balloon-expandable Edwards Sapien or Sapien XT systems [20]. In our study, the incidence of LBBB, stratified by the type of valve deployed, was as follows: Abbott Vascular Portico/Navitor (23.9%), Myval (23.1%), Medtronic Evolut R/Pro (11.5%), Acurate Neo 2 (8.6%), and Sapien XT/S3 (8.2%).

One of the most investigated outcomes post-TAVR is the need for a new PPI [5]. Studies from developed countries have reported post-TAVR PPI rates ranging between 9% and 26%. In our study, the PPI rate was relatively low at discharge (5.8% or 23 patients) and at 30 days (7% or 28 patients). Bahaa et al. reported a similar overall PPI requirement of 7.29% in their Egyptian cohort, which aligns closely with our findings, demonstrating comparable procedural outcomes across different settings [14]. Multiple factors were associated with an increased risk of PPI post-TAVR, which can be divided into pre- and intra-procedural characteristics. Pre-procedural risk factors include male sex, baseline first-degree AV block, left anterior hemiblock, right bundle-branch block (RBBB), QRS prolongation (>120 ms), and calcifications involving the aortic valve, LVOT, and membranous septum length [21]. Intra-procedural predictors include the presence of intraoperative heart block, depth of prosthesis implantation, valve oversizing, insufficient difference between membranous septum length and depth of implantation, and the use of a self-expandable prosthesis.

This low rate of new pacemaker implantation in our population might have been influenced by the techniques our operators have adopted. These included high implantation using the lucent line for balloon-expandable valves (Edwards Sapien), the cusp overlap technique for self-expanding valves (Medtronic Evolut R/Pro), and precise measurement of the membranous septum length in most of our patients. Throughout the study period, 78.5% of PPIs were due to third-degree AV block, followed by LBBB (10.7%), SSS (7.1%), and trifascicular block (3.6%). The highest rate of PPI was recorded in the high-risk group at discharge and at 30 days post-procedure (11.1% or seven patients). Among high-risk patients with PPI post-TAVR, 20% had baseline RBBB, 70% underwent pre-dilation, and 30% underwent post-dilation-factors reported to be associated with a higher risk of PPI [18]. A higher rate of PPI in TAVR patients receiving self-expandable valves (such as Medtronic CoreValve) compared to those receiving a balloon-expandable valve (such as Edwards Sapien/Sapien XT valve) has been reported in the literature. However, in our study, the rate of PPI was not influenced by the choice of bioprosthetic valve deployed.

PVL is a common adverse event post-TAVR, with an overall incidence ranging between 50% and 85% [22]. In our study, a lower PVL rate of 41.7% (165 patients) was recorded, of which 72.7% (120 patients) were grade 1, 22.4% (37 patients) grade 2, 4.25% (seven patients) grade 3, and 0.6% (one patient) grade 5. This lower rate can be attributed to the thorough evaluation of the aortic valve by CT scan and correct sizing of the annulus. The tailored approach to bioprosthetic valve selection and the use of new-generation devices equipped with sealing skirts were instrumental in achieving these results.

Different approaches regarding access sites for TAVR are available. The preferred and most commonly used access site remains the femoral one [23]. Alternative approaches include transapical, transaortic, transcaval, transcarotid, and transaxillary; however, these are now more limited due to the availability of lower-profile delivery systems and the use of shockwave balloons. Vascular and access-related complications were quite common with early-generation TAVR devices, ranging between 10% and 20% in developed countries [23]. In this study, a lower rate of 6.6% (26 patients) was recorded, of which 11.5% (three patients) were major cases.

The healthcare system in Lebanon is a mix of private and public. There is a public health sector that provides some basic services to citizens, but it is often inadequate and underfunded. As a result, many people opt to pay for private healthcare services, which are generally of higher quality. The mix of private and public healthcare in Lebanon has resulted in a fragmented and unequal system, with access to care and quality of care varying greatly depending on a person's income and resources. In recent years, Lebanon has faced numerous challenges including economic crises, political instability, and an influx of refugees from neighboring countries [24,25]. These factors have put an additional strain on the country's healthcare system, leading to shortages of medical supplies and personnel, as well as reduced access to quality medical care for many individuals [26].

The AUBMC is a private healthcare center known for its state-of-the-art facilities and high-quality medical care and is one of the leading healthcare providers in the region. Patients at AUBMC typically pay for their medical care out of pocket, with private health insurance, and public coverage through the National Social Security Fund. However, the cost of a novel and complex procedure like TAVR is still considered a high burden to our candidates, rendering it less attainable than other medical and surgical alternatives. Nevertheless, despite these challenges, the structural heart program at AUBMC was able to provide top-notch patient care evidenced by our highly favorable results.

TAVR clinical outcomes at our center were found comparable to the biggest international healthcare centers which in turn highlights the importance of a well-structured heart team program. The program comprised interventional cardiologists, cardiovascular surgeons, registered nurses, and a TAVR coordinator whose aim was to provide the best possible care to TAVR patients by following a number of best practices and guidelines.

Multiple limitations to this study ought to be mentioned. First, the single center setting of this study and its retrospective nature may be restrictive of the data randomization and study impact. However, the population represented is by consequence homogenous with the consistent quality of results interpretation and clinical outcomes. Second, the follow-up duration is limited to 30 days post-procedure in this report, discounting the evaluation of valve durability and long-term efficacy. Long-term data should be analyzed when available. Although patients were stratified according to their risk score, the STS score is suboptimal as proven by multiple other studies [27]. Frailty scores, porcelain aorta, and other factors are not integrated into the risk scores which is a major limiting factor to adequate risk assessment. Moreover, due to the small sample size, we were not able to conduct further analysis such as multivariable logistic regression, thus our analysis did not consider the effect of possible confounders on our results. Nevertheless, TAVR has become the alternative management for all surgical-risk patients and it is worth reporting its outcomes for the whole.

## Conclusions

This study underscored the successful implementation of the TAVR program at AUBMC, demonstrating high rates of procedural success and favorable early safety profiles within the initial 30 days post-operation. Despite the notable challenges of financial constraints, limited specialized training, and disparities in healthcare access in developing countries, this study highlighted the potential of TAVR as a viable and effective treatment for patients with severe AS across different risk profiles. Large cohorts and longer follow-up durations are needed to accurately represent developing countries' TAVR clinical outcomes and consequently TAVR global data. Moreover, addressing the medical shortages and issues developing countries face requires collaborative efforts between international organizations, governments, and healthcare institutions. Additionally, knowledge-sharing initiatives, partnerships with developed countries, and research collaborations can help bridge the gap between developed and developing nations, ensuring that patients worldwide have equal opportunities to benefit from the advancements in TAVR and cardiovascular care.
